# Supplementary material for: The Composition of Fatty Acids in Bee Pollen, Royal Jelly, Buckthorn Oil and Their Mixtures with Pollen Preserved for Storage
Source: Foods. 2023 Aug 23;12(17):3164. doi: 10.3390/foods12173164 (PMC10487168; doi:10.3390/foods12173164)
Supplement: Supplementary file 1 [file foods-12-03164-s001.zip › foods-2534425-supplementary.pdf]

Supplementary Material

Table S1. Comparative average of fatty acids (FA) content (% of total FA) of pollen preserved with honey and their mixtures with sea buckthorn oil (SBO) and royal jelly (RJ) in a two-year period of storage at +4 °C ($p \leq 0.05$).

Fatty acids	Formula	BPH	BPH +1 % SBO	BPH +1% SBO +2 % RJ	*P* < 0.05 value
Lauric	C12:0	0.20	0.14	0.13	0.03
Myristic	C14:0	1.51	0.91	0.94	0.10
Pentadecanoic	C15:0	0.41	0.22	0.23	0.26
Palmitic	C16:0	23.85	19.19	19.30	1.07
Palmitoleic	C16:1n-7	0.74	3.68	3.58	0.38
Stearic	C18:0	2.32	2.51	2.55	0.23
Oleic	C18:1n-9*cis*	6.26	8.46	8.34	0.83
Vaccenic	C18:1n-7	1.62	1.85	1.92	0.16
Linolelaidic	C18:2n6*trans*	0.81	0.68	0.70	0.45
Linoleic	C18:2n-6*cis*	10.48	28.78	27.99	2.06
γ-Linoleic	C18:3n-6	1.16	0.99	0.96	0.24
α-Linolenic	C18:3n-3	42.31	26.52	27.41	0.83
Arachidic	C20:0	0.64	0.56	0.54	0.10
Eicosenoic	C20:1n-9	0.15	0.16	0.15	0.06
Eicosadienoic	C20:2n-6	0.19	0.15	0.17	0.05
Eicosatrienoic	C20:3n-3	0.41	0.24	0.25	0.07
Arachidonic	C20:4n-6	0.61	0.16	0.13	0.57
Behenic	C22:0	0.35	0.45	0.46	0.04
Docosadienoic	C22:2n-6	0.31	0.15	0.11	0.29
Docosatetraenoic	C22:4n-6	0.23	0.21	0.22	0.12

Lignoceric	C24:0	0.31	0.23	0.26	0.07
Nervonic	C24:1n-9	0.43	0.30	0.28	0.08

Note: Saturated fatty acids (SFA): C12:0; C14:0; C15:0; C16:0; C18:0; C20:0; C22:0; C24:0

Unsaturated fatty acids (USFA): n-3; n-6; n-7; n-9; C24:1

n-3: C18:3n3; C20:5n3 and n-6: C18:2n6*trans*; C18:2n6*cis*; C18:3n6; C20:2n6; C20:4n6; C22:2n6; C22:4n6

n-7: C16:1n7; C18:1n7 and n-9: C18:1n9*cis*; C20:1n9; C24:1n9

The bee products used in the study included pollen mixed with honey in ratio 1:2 g/g represented as BPH, pollen mixed with honey in ratio 1:2+1 % (*w/w*) SBO indicated as BPH+1 % (*w/w*) SBO), pollen mixed with honey in ratio 1:2+1 % (*w/w*) SBO+2 % RJ indicated as BPH+1% (*w/w*) SBO+2 % (*w/w*) RJ)

Table S2. Basic statistics and relationship of fatty acids (% of total FA) between bee pollen and honey mixture with pollen during a two-year period of storage at +4 °C ($p \leq 0.05$).

Fatty acids	Formula	BP, %				BPH, %			(*r*)	CV
		Avg±SE	Min	Max	CV	Avg±SE	Min	Max		
Lauric	C12:0	0.32±0.07	0.21	0.53	44.7	0.20±0.01	0.16	0.23	0.76	14.7
Myristic	C14:0	1.53±0.12	1.20	1.75	15.5	1.51±0.09	1.28	1.72	0.82	11.9
Pentadecanoic	C15:0	0.57±0.16	0.12	0.88	56.4	0.41±0.21	0.09	0.96	0.78	1013
Palmitic	C16:0	23.31±0.52	21.91	24.31	4.43	23.85±0.33	22.88	24.39	-<br>0.36	2.78
Palmitoleic	C16:1n-7	0.18±0.01	0.16	0.22	15.05	0.74±0.47	0.19	2.16	0.99	127.6
Stearic	C18:0	1.97±0.16	1.68	2.36	16.36	2.32±0.10	2.14	2.56	-<br>0.28	8.82
Oleic	C18:1n-9 *cis*	6.36±0.66	4.85	7.94	20.69	6.26±0.51	5.03	7.45	0.39	16.16
Vaccenic	C18:1n-7	1.56±0.19	1.22	2.04	23.77	1.62±0.24	1.27	2.34	0.88	30.14
Linolelaidic	C18:2n-6*trans*	1.04±0.42	0.19	2.10	80.31	0.81±.33	0.15	1.50	0.93	82.05
Linoleic	C18:2n-6*cis*	9.77±0.19	9.40	10.16	3.95	10.48±0.34	9.76	11.32	-<br>0.47	6.43
γ-Linoleic	C18:3n-6	0.80±0.55	0.21	2.45	136.9	1.16±0.55	0.18	2.11	0.59	94.10
α-Linolenic	C18:3n-3	41.36±1.26	38.59	43.94	6.11	42.31±0.72	40.24	43.57	-<br>0.59	3.42
Arachidic	C20:0	0.75±0.05	0.65	0.88	13.35	0.64±0.06	0.54	0.77	0.98	17.26
Eicosenoic	C20:1n-9	1.13±0.58	0.17	2.58	102.1	0.15±0.01	0.12	0.18	-<br>0.48	16.69
Eicosadienoic	C20:2n-6	0.22±0.02	0.17	0.28	20.23	0.19±0.03	0.12	0.23	0.27	26.14
Eicosatrienoic	C20:3n-3	0.65±0.13	0.28	0.90	40.95	0.41±0.04	0.31	0.51	0.94	20.01
Arachidonic	C20:4n-6	0.49±0.12	0.35	0.74	43.49	0.61±0.39	0.18	1.40	0.99	111.3
Behenic	C22:0	0.44±0.07	0.32	0.62	31.72	0.35±0.02	0.32	0.40	0.98	9.62
Docosadienoic	C22:2n-6	0.38±0.13	0.24	0.67		0.31±0.02	0.29		0.99	51.67<br>6.96
Docosatetraenoic	C22:4n-6	0.28±0.04	0.18	0.35		0.23±0.06	0.14		0.43	26.64<br>51.38
Lignoceric	C24:0	0.23±0.06	0.09	0.33		0.31±0.02	0.25		0.90	47.69<br>14.87
Nervonic	C24:1n-9	0.35±0.04	0.25	0.44		0.43±0.04	0.35		0.31	23.68<br>16.22

Note: Saturated fatty acids (SFA): C12:0; C14:0; C15:0; C16:0; C18:0; C20:0; C22:0; C24:0

Unsaturated fatty acids (USFA): n-3; n-6; n-7; n-9; C24:1

n-3: C18:3n3; C20:3n3 and n-6: C18:2n6*trans*; C18:2n6*cis*; C18:3n6; C20:2n6; C20:4n6; C22:2n6; C22:4n6

n-7: C16:1n7; C18:1n7 and n-9: C18:1n9*cis*; C20:1n9; C24:1n9

AVG – average, CV – coefficient of variation and SE – standard error

The bee products used in the study included pollen mixed with honey in ratio 1:2 g/g represented as BPH
